# Construction of High-Density Linkage Maps of *Populus deltoides* × *P*. *simonii* Using Restriction-Site Associated DNA Sequencing

**DOI:** 10.1371/journal.pone.0150692

**Published:** 2016-03-10

**Authors:** Chunfa Tong, Huogen Li, Ying Wang, Xuran Li, Jiajia Ou, Deyuan Wang, Houxi Xu, Chao Ma, Xianye Lang, Guangxin Liu, Bo Zhang, Jisen Shi

**Affiliations:** The Southern Modern Forestry Collaborative Innovation Center, College of Forestry, Nanjing Forestry University, Nanjing, 210037, China; Universidad Miguel Hernández de Elche, SPAIN

## Abstract

Although numerous linkage maps have been constructed in the genus *Populus*, they are typically sparse and thus have limited applications due to low throughput of traditional molecular markers. Restriction-site associated DNA sequencing (RADSeq) technology allows us to identify a large number of single nucleotide polymorphisms (SNP) across genomes of many individuals in a fast and cost-effective way, and makes it possible to construct high-density genetic linkage maps. We performed RADSeq for 299 progeny and their two parents in an F_1_ hybrid population generated by crossing the female *Populus deltoides* ‘I-69’ and male *Populus simonii* ‘L3’. A total of 2,545 high quality SNP markers were obtained and two parent-specific linkage maps were constructed. The female genetic map contained 1601 SNPs and 20 linkage groups, spanning 4,249.12 cM of the genome with an average distance of 2.69 cM between adjacent markers, while the male map consisted of 940 SNPs and also 20 linkage groups with a total length of 3,816.24 cM and an average marker interval distance of 4.15 cM. Finally, our analysis revealed that synteny and collinearity are highly conserved between the parental linkage maps and the reference genome of *P*. *trichocarpa*. We demonstrated that RAD sequencing is a powerful technique capable of rapidly generating a large number of SNPs for constructing genetic maps in outbred forest trees. The high-quality linkage maps constructed here provided reliable genetic resources to facilitate locating quantitative trait loci (QTLs) that control growth and wood quality traits in the hybrid population.

## Introduction

The *Populus* genus not only has several attractive biological characteristics as a long-lived plant but also possesses tremendous economic and ecological importance. Because of its fast growth, asexual reproduction, small genome size (~480 Mbp) and easy genetic transformation, this genus was selected as a model system for forest trees [1, 2] and the species of *P*. *trichocarpa* and *P*. *euphratica* have had their genomes sequenced successively [3, 4]. Some species of *Populus* are cultivated worldwide to meet demand for pulp and paper, lumber, wood-based panels, and biofuels. Others are planted on a large scale to build windbreaks especially in Northwest China where sand-dust storms occur every year. The *Populus* genus comprises aspens, cottonwoods and poplars, and there are approximately 30 species that can be grouped into six separate sections [5]. The overwhelming majority of these species have an extensive distribution in the Northern Hemisphere, and harbour significant variability in adaptive traits such as growth rate, rooting ability, and drought or disease resistance. Nevertheless, there is an increasing interest in creating new varieties for superior adaptive and commercial traits through intraspecific or interspecific hybridization. High-quality genetic linkage maps provide valuable genomic information for achieving this goal by mapping quantitative trait loci (QTLs) and marker-assisted selection.

In the past two decades, great efforts have been made to develop numerous genetic linkage maps of different *Populus* species. Many of these linkage maps were constructed directly using the software MAPMAKER [6] with mapping strategies of inbred lines, including so-called ‘pseudo-testcross’ proposed by Grattapaglia and Sederoff [7–19]. Only a few studies have constructed *Populus* linkage maps with the software JoinMap [20], although this tool can incorporate different linkage phases and various segregation markers into linkage analyses for an outbred population [21–24]. All these maps were mainly constructed with molecular markers such as RAPD, RFLP, AFLP and SSR. However, these traditional molecular markers are of low throughput due to instability or time- and cost-consuming experiments, and hence cannot satisfy the needs of constructing high-density genetic linkage maps in forest trees. To date, the densest linkage map of *P*. *trichocarpa* contains more than 3,500 BeadArray SNP markers, which was reported in Slavov et al. [25] and again in Muchero et al. [26].

Restriction-site associated DNA sequencing (RADSeq), one of the next generation sequencing (NGS) technologies based at reduced genome complexity [27], allows for tens of thousands of single nucleotide polymorphisms (SNPs) to be identified in a fast and relatively cheap way across the genomes of many individuals from an experimental population. This powerful new sequencing method was first described by Baird et al. [28], where more than 13,000 SNPs were identified and used to map three traits in two model organisms, threespine stickleback and *Neurospora crassa*. Later on, Emerson et al. [29] used RADSeq technology to reveal an evolutionary mystery in the pitcher plant mosquito, *Wyeomyia smithii*. In the meantime, Hohenlohe et al. [30] applied RADSeq method to study parallel adaption in natural populations of threespine stickleback. Recently, a series of linkage maps constructed with RADSeq markers were reported in various organisms, including ryegrass, barley, moth, grape and gudgeon [31–35]. These RADSeq genetic linkage maps generally included more than 1,000 molecular markers with an average distance of less than 5 cM between adjacent markers, and hence provided reliable genetic resources for population genetics in related species.

The objective of this study was to construct high-density and high-quality genetic linkage maps of *Populus* with RADSeq technology for extensive QTL mapping studies in the future. An interspecific cross was conducted to generate an F_1_ mapping population by hybridizing *Populus deltoides* with *P*. *simonii*. RADSeq was performed using genomic DNA from the two parents and 299 progeny. A large number of SNPs that follow Mendel’s segregation ratio were identified and genotyped through mapping RAD reads of each individual to the reference genome of *P*. *trichocarpa* as well as some rigorous filtering procedures. Based on these SNP molecular markers, the maternal and paternal genetic linkage maps were constructed separately with linkage phase inferred between adjacent markers. The synteny and collinearity between the linkage maps and the reference genome were evaluated and were mostly consistent, indicating that the linkage maps are highly accurate in marker ordering. The dense and reliable linkage maps of the two parents are useful for identifying QTLs that control growth and timber traits in the permanent *Populus* population.

## Materials and Methods

### The Mapping Population and DNA Isolation

The mapping population was generated by hybridizing *P*. *deltoides* Marsh cv. ‘Lux’ (I-69/55) with *P*. *simonii* in 2011. *P*. *deltoides* has the superior characteristics of fast growth and resistance to *Marssonina* leaf spot disease, but has a low survival rate of field cutting propagation due to the poor rooting ability [17]. *P*. *simonii*, a native tree species widely distributed in northern China, displays excellent performance in cold, heat and drought, tolerance to alkali-salt and barren conditions, and regeneration ability [36]. These two parental *Populus* could produce hybrids with significant segregation of morphological and physiological traits. The female *P*. *deltoides* ‘I-69’ was chosen from Siyang Forest Farm (SFF) of Siyang County, Jiangsu Province, China, while the flowering branches of *P*. *simonii* ‘L-3’ were collected in a forest land managed by Luoning Forest Bureau of Henan Provicne, China. The interspecific cross was performed in the spring of 2011 in SFF. Approximately 500 seedlings of the F_1_ progeny were planted in Xiashu Forest Farm of Nanjing Forest University, Jurong County, Jiangsu Province, China. A total of 299 progeny were randomly chosen for constructing linkage maps of the two parents in this study. Young leaf tissue was collected from the two parents and each of the 299 individuals at the beginning of the vegetative period (late spring). The samples were immediately stored in a -80°C freezer. DNA was extracted from 150 individuals using the Plant Genomic DNA Kit (Tiangen, Tiangen Biotech Co. Ltd., Beijing) with the manufacturer's protocol, while the other 149 individuals DNA were extracted with the CTAB protocol [37].

This study was permitted to conduct in Luoning County of Henan province, China, and Siyang County of Jiangsu province, China, by the two local Agriculture and Forestry Commissions, and in Xiashu Forest Farm of Nanjing Forestry University located in Jurong County, Jiangsu province, China, by the Academic Committee of the university. All the test fields belong to the local government or university and no endangered or protected species were involved in our study.

### RAD Sequencing

The RAD library was constructed following the protocol described by Baird et al. [28]. Genomic DNA from each of the two parents (1.5 μg) and 299 progeny (300 ng) was digested for 4 h at 37°C in a 50-μL reaction with 20 units (U) of *EcoRI* (NEB, USA). The reactions were stopped by holding at 65°C for 20 min. 200 nM P_1_ adapter was ligated to the products of the restriction reaction, along with 5 μL of 10 mM ATP, 1 μL 10x NEB Buffer 2, 0.5 μL (1,000 U) T4 DNA Ligase (high concentration, NEB) and 5 μL H2O, and incubated at room temperature for 20 min. The P_1_ adapter, a modified Solexa adapter (top: 5’-CAAGCAGAAGACGGCATACGAGATXXXX XXGTGACTGGAGTTCAGACGTGTGCTCTTCCGATCT-3’, bottom: 3’-GTTCG TCTTCTGCCGTATGCTCTAXXXXXXCACTGACCTCAAGTCTGCACACGAG AAGGCTAGATTAA-p-5’, X indicated MID), contained a matching sticky-end to the fragments and a MID (Molecular Identifier), a short sequence that will uniquely identify an individual. Samples were again heat-inactivated for 20 min at 65°C, pooled and purified with a Qiagen PCR cleanup column and eluted in 30 μL of buffer EB. DNA was sheared using Covaris S220 into 300–700 bp. Fragment ends were repaired using the Quick Blunting kit Enzyme Mix (NEB) with 30 μL DNA, 5 μL 10x Blunting Buffer, 10 μL 1Mm dNTP and 9 μL H2O, incubated at 25°C for 30 min, purified with 1.6 times the volume of AMPure XP Beads and eluted in 21 μL water. dATP overhangs were added to the DNA using 20 μL of purified library template, dATP (1 μL 100 mM), 15U of Klenow exo- (NEB) and 3 μL 10x NEB Buffer 2. The reaction was incubated at 37°C for 30min, then purified with 1.6 times the volume of AMPure XP Beads column and eluted in 21 μL water.

Paired-end P2 adapter (top: 5’-AATGATACGGCGACCACCGAGATCTAC ACTCTTTCCCTACACGACGCTCTTCCGATCT-3’, bottom: 3’-TTACTATGCCG CTGGTGGCTCTAGATGTGAGAAAGGGATGTGCTGCGAGAAGGCTAG-p-5’), a divergent modified Solexa adapter (2006 Illumina, Inc.), was ligated to 20 μL sheared, size-selected, P1-ligated and pooled DNA template with 2 μL of 2 μM Adapter2, 3 μL of NEB Buffer 2 and 1 μL of 1,000 U T4 DNA Ligase in total reaction of 30 μL. The ligation was incubated overnight at 4°C, then DNA product purified with a same volume of AMPure XP Beads and eluted in 30 μL of water. PCR enrichment of the library was performed in a PCR amplification with 25 μL 2x Phusion PCR Master Mix (NEB), 1.5 μL of PCR Primer 1: 5'-AATGATA CGGCGACCACCGA-3', 1.5 μL of PCR Primer 2: 5'-CAAGCAGAAGACGGC ATACGAG-3' and 14 μL of H2O. Cycling conditions were: 98°C for 1 min, then 18 cycles of 98°C for 10 s, 60°C for 30 s, 72°C for 40 s, and a final extension at 72°C for 5 min. PCR amplicons were gel purified and fragments in the size range 300–700 bp were excised from the gel.

The RAD library was sequenced using an Illumina HiSeq 2000 sequencer. The two parents and one half of the progeny were sequenced in 8 lanes (paired-end, 100 bp) at Novogene Bioinformatics Institute (NBI), Beijing, China. In addition, the two parents and another half of the progeny were sequenced in 7 lanes (paired-end, 90 bp) at Beijing Genomics Institute (BGI), Shenzhen, China.

### RAD Sequence Analysis, SNP Discovery and Genotyping

Standard quality control (QC) pipelines (NBI, Beijing, China; BGI, Shenzhen, China) were used to process the raw sequencing data. First, multiple sequences were segregated by the appropriate nucleotide MID assigned to each sample, and paired reads containing primer/adaptor sequence were removed. Second, when one single read contains more than 10% of its bases uncalled, the pair was discarded. Third, if the number of low-quality bases (Q score less than or equal to 5) was greater than 50% of the length in a single read, then both of the paired reads were also removed from the dataset. After these filtering procedures, the so-called clean data reads were generated for each sample. We further used NGS QC toolkit (v2.3.3, [38]) with the default cut-off values to filter these clean data and to obtain high-quality (HQ) reads that each has at least 70% bases with the Phred quality score more than or equal to 20.

The filtered HQ reads were mapped to the reference genome of *P*. *trichocarpa* [3] (V3.0, DOE-JGI, http://www.phytozome.net/poplar), which consists of approximately 434.1 Mb arranged into 19 primary scaffolds (corresponding to the 19 chromosomes, 394.5 Mb) and 1427 additional scaffolds. First, we used the BWA [39] *mem* command with default parameters to align the paired-end reads of each sample separately to the reference sequences. Subsequently, a sequence alignment/map (SAM) format file for each individual was produced [40]. Next, several steps were taken for SNP calling and genotyping with SAMtools (including bcftools, [40]) and Perl scripts (available upon request): (i) filtering out records in each SAM file, which have multiple mapping positions on the genome; (ii) producing BCF files with command *samtools mpileup–g–f -I*; (iii) generating the variant call format (VCF, [41]) files with command *bcftools view–c–v* for each parent; (iv) filtering SNPs with relatively relaxed conditions (quality more than 20 and DP more than or equal to 5) for each parent using Perl scripts, merging the SNP data sets and saving all parental SNP positions as a list site file used in the next step; (v) for all progeny, filtering SAM files as step (i), generating BCF files as step (ii), and creating VCF files with command *bcftools view–l–c–g* using the list site file; (vi) identifying genotype of each progeny according to the genotype likehihoods (GL) of all possible genotypes at each SNP in VCF files, and filtering with stringent conditions (DP more than or equal to 10 and GQ more than 50). Finally, we further filtered those SNPs for linkage mapping, which followed Mendelian segregation ratios by chi-square test across the whole population.

### Linkage Map Construction

We performed chi-square tests with different degrees of freedom to check whether the genotyped SNPs follow the corresponding Mendelian segregation ratios, such as 1:1, 1:2:1 and 1:1:1:1. Those SNPs deviating seriously from the Mendelian ratios (*p <* 0.01) and having more than 10% missing genotypes in the population were removed from linkage analysis. Since the overwhelming majority of SNPs segregate in 1:1 in this study, the pseudo-testcross mapping strategy [7] was applied to construct two linkage maps, each for a different parent. The construction of both parental linkage maps was carried out with two softwares, JoinMap 4.1 [20] and FsLinkageMap 2.1 [42]. The maternal linkage map was constructed using the SNP markers that have the segregation type of *abaa*, where *ab* represents the heterozygous genotype from the mother and *aa* the homozygous genotype from the father, while the paternal linkage map was built with the markers of segregation type of *aaab*. First, two-point linkage analysis was conducted and linkage groups were clustered under the threshold of LOD score using FsLinkageMap. Second, markers in each linkage group were ordered three times using the maximum likelihood (ML) mapping algorithm with default parameters in JoinMap and one time using the ordering method in FsLinkageMap. The best order was chosen as the mapping order among the four results of the two programs, based on the minimum sum of adjacent recombination fractions (SARF; [43]). Third, the linkage maps were drawn in WMF format with FsLinkageMap and then edited in PDF or EPS format with the software of Mayura Draw (http://www.mayura.com).

### QTL Mapping

We measured each tree height and diameter at breast height (DBH) in the fall of 2014 and performed QTL mapping for the two growth traits based on the two parental linkage maps constructed here. The mapping method is a modified composite interval mapping (CIM; [44]) that can incorporate different segregation types of markers and various linkage phases. The log likelihood ratio (LR) at each map position was calculated with 15 background markers selected by forward regression method and a window size of 10 cM. The threshold value for asserting the existence of a QTL was determined by 1000 permutation tests [45]. The algorithm was implemented with in-house R scripts, which are available upon request.

## Results

### RAD Sequencing and SNP Genotype Calling

A total of 329.5 Gb raw RADSeq data containing 1,790,149,605 paired-end (PE) reads was generated on the Illumina HiSeq2000 (Table 1). Of these data, 142.0 Gb of 100-bp PE reads for the two parents and 150 progeny were produced from the sequencing experiment performed in NBI, while the rest of 187.5 Gb 90-bp PE reads for the same two parents and other 149 progeny was produced by sequencing in BGI. The RADSeq raw data are available under accession number SRP052929 at the NCBI Sequence Read Archive database (http://www.ncbi.nlm.nih.gov/Traces/sra). After a series of quality control and filtering procedures (described in Materials and Methods), we obtained 309.3 Gb of HQ reads. The female *P*. *deltoides* parent yielded 5.9 Gb of HQ reads (2.3 Gb from NBI, 3.6 Gb from BGI), whereas the male *P*. *simonii* parent yielded 12.2 Gb of HQ reads (2.5 Gb from NBI, 9.7 Gb from BGI). On average, 0.8 Gb of HQ 100-bp PE reads was obtained for the 150 progeny sequenced in NBI and 1.1 Gb of HQ PE reads for the additional 149 progeny sequenced in BGI. It is worthy of note that one sample with field ID, ‘C15-1’, as well as the two parents, were independently sequenced in both sequencing companies and can be used to confirm the accuracy of sequencing. The length of the HQ forward reads generated in BGI ranged from 82 to 86 bp due to discarding the original 4–8 bp MID barcode sequences that identify individual samples within a pooled library. All these RADSeq data of HQ PE reads allowed for calling SNP genotypes across the genomes of the two parents and 299 progeny in the full-sib family of *P*. *deltoides* × *P*. *simonii*.

**Table 1:** Summary of RADSeq Data from NBI and BGI with averages in brackets.

Experiment	Sample	No. sample	No. raw reads	Raw reads data (Gb)	No. HQ reads	HQ reads data (Gb)
NBI	Male parent	1	14,024,713	2.80	12,617,155	2.52
	Female parent	1	12,949,974	2.59	11,500,364	2.30
	Progeny	150	683,250,607 (4,555,004)	136.65 (0.91)	612,650,551 (4,084,337)	122.53 (0.82)
BGI	Male parent	1	57,159,139	9.91	55,789,694	9.68
	Female parent	1	21,619,787	3.72	20,956,868	3.60
	Progeny	149	1,001,145,385 (6,719,097)	173.87 (1.17)	971,115,096 (6,517,551)	168.66 (1.13)
Total	Male parent	1	71,183,852	12.72	68,406,849	12.20
	Female parent	1	34,569,761	6.31	32,457,232	5.90
	Progeny	299	1,684,395,992	310.52	1,583,765,647	291.19
	Total	301	1,790,149,605 (5,947,341)	329.55 (1.10)	1,684,629,728 (5,596,777)	309.29 (1.03)

With the short read mapping program, BWA, 94.1% of the HQ reads from the female parent and the same ratio from the male parent were mapped to the *Populus* reference genome. However, only 23.6% of the maternal HQ reads each was aligned to the reference genome with the edit distance less than or equal to 8, the alignment score more than 60, and the alignment score less than 10 for second-best alignment, while for the male parent this fraction is nearly the same (23.1%) as the female parent. On average, these almost uniquely mapped HQ reads reached 31.24-fold and 61.66-fold coverage depth, and covered 8.98% and 9.14% of the reference genome for the female and male parents, respectively. For all the progeny, the average coverage depth of such reads ranged from the minimum of 3.54-fold to the maximum of 21-fold and the coverage of the reference genome from 4.96% to 8.83% (S1 Table). We used the almost uniquely mapped HQ reads from each parent for SNP calling. As a result, 836,895 SNPs with depth of more than or equal to 5 were revealed in the two parents, of which 475,965 SNPs were from the female, 475,591 SNPs from the male, and 114,661 SNPs from both. We further performed SNP genotype calling for the parents and progeny within those SNPs identified in the two parents. We filtered out those SNPs at which one parent has genotype, but the other does not due to no or low coverage of reads. Consequently, there were 385,470 SNPs genotyped in both parents; however, only 85,363 SNPs segregated in the progeny, with segregation patterns of *abaa* (51,817), *aaab* (32,654), *abab* (793), and *abac* (99) (Table 2). Finally, 1,603 and 942 SNPs with segregation types of *abaa* and *aaab*, respectively, were chosen for linkage mapping, which followed the Mendelian segregation ratio 1:1 with *p* ≥ 0.01 and at which at least 90% of the 299 progeny were genotyped. The distance of adjacent SNPs with same segregation patterns is more than 1 kb on the reference genome.

**Table 2:** Number of SNP loci genotyped in both parents and used for linkage mapping.

Female genotype^a^	Male genotype	SNPs in parents	SNPs used for mapping
*ab*	*aa*	51,817	1,603
*aa*	*ab*	32,654	942
*ab*	*ab*	793	0
*ab*	*ac*	99	0
*aa*	*bb*	300,107	0
Total		385,470	2,545

^*a*^The notations of *a*, *b*, *c* and *d* denote up to four possible alleles from two parents at an SNP site.

To validate the accuracy of SNP genotypes, we used the two RADSeq data sets from NBI and BGI to independently call genotypes for the two parents, ‘I-69’ and ‘L-3’, and one progeny, ‘C15-1’. Of the 2545 SNPs for linkage mapping, over 97.0% were genotyped with the two data sets for each of the 3 samples. Among those SNPs genotyped with the two data sets for each sample, ~98% were confirmed with each other’s data set, resulting in totally 98.2% of the 7496 SNP genotypes confirmed for all the 3 samples (S2 Table).

### Genetic Linkage Maps and QTL Mapping

**Fig 1:**
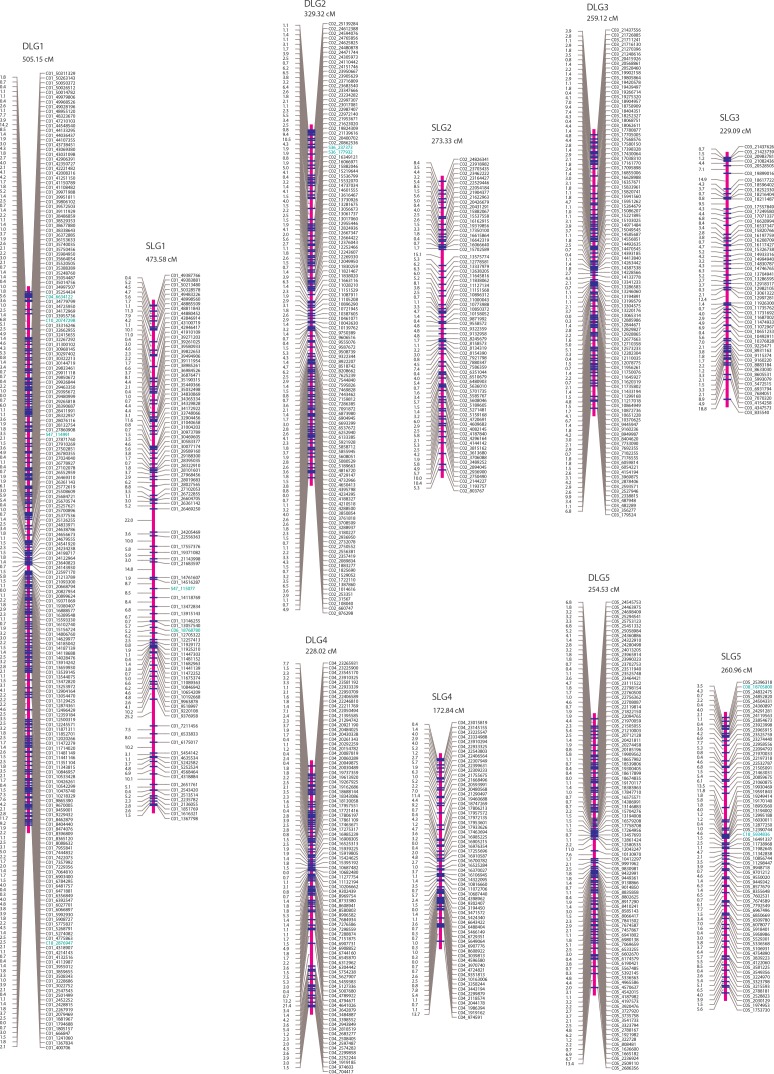
The genetic maps of linkage groups DLG1-DLG5 for the maternal *P*. *deltoides* ‘I-69’ and SLG1-SLG5 for the paternal *P*. *simonii* ‘L-3’. The length of each linkage group is given below the linkage group name. SNP marker names are denoted by the names and positions of chromosomes or scaffolds of the reference genome *P*. *trichocarpa*. Markers from other chromosomes and additional scaffolds are in green.

**Fig 2:**
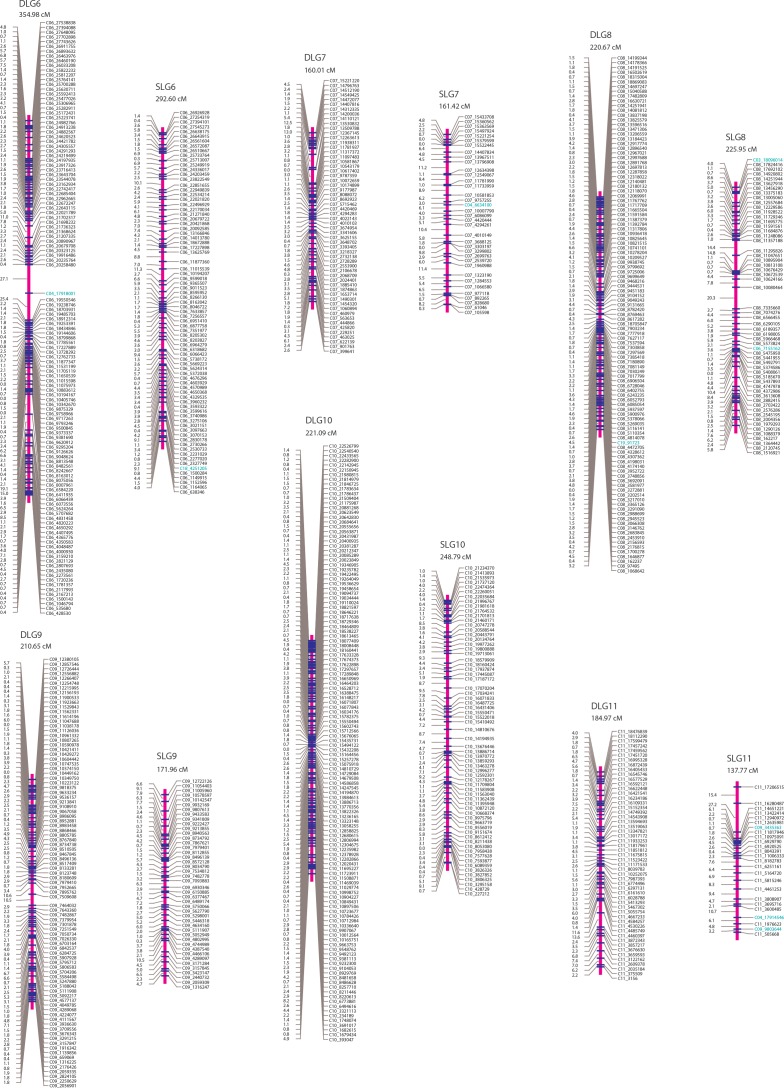
The genetic maps of linkage groups DLG6-DLG11 for the maternal *P*. *deltoides* ‘I-69’ and SLG6-SLG11 for the paternal *P*. *simonii* ‘L-3’. The length of each linkage group is given below the linkage group name. SNP marker names are denoted by the names and positions of chromosomes or scaffolds of the reference genome *P*. *trichocarpa*. Markers from other chromosomes and additional scaffolds are in green.

**Fig 3:**
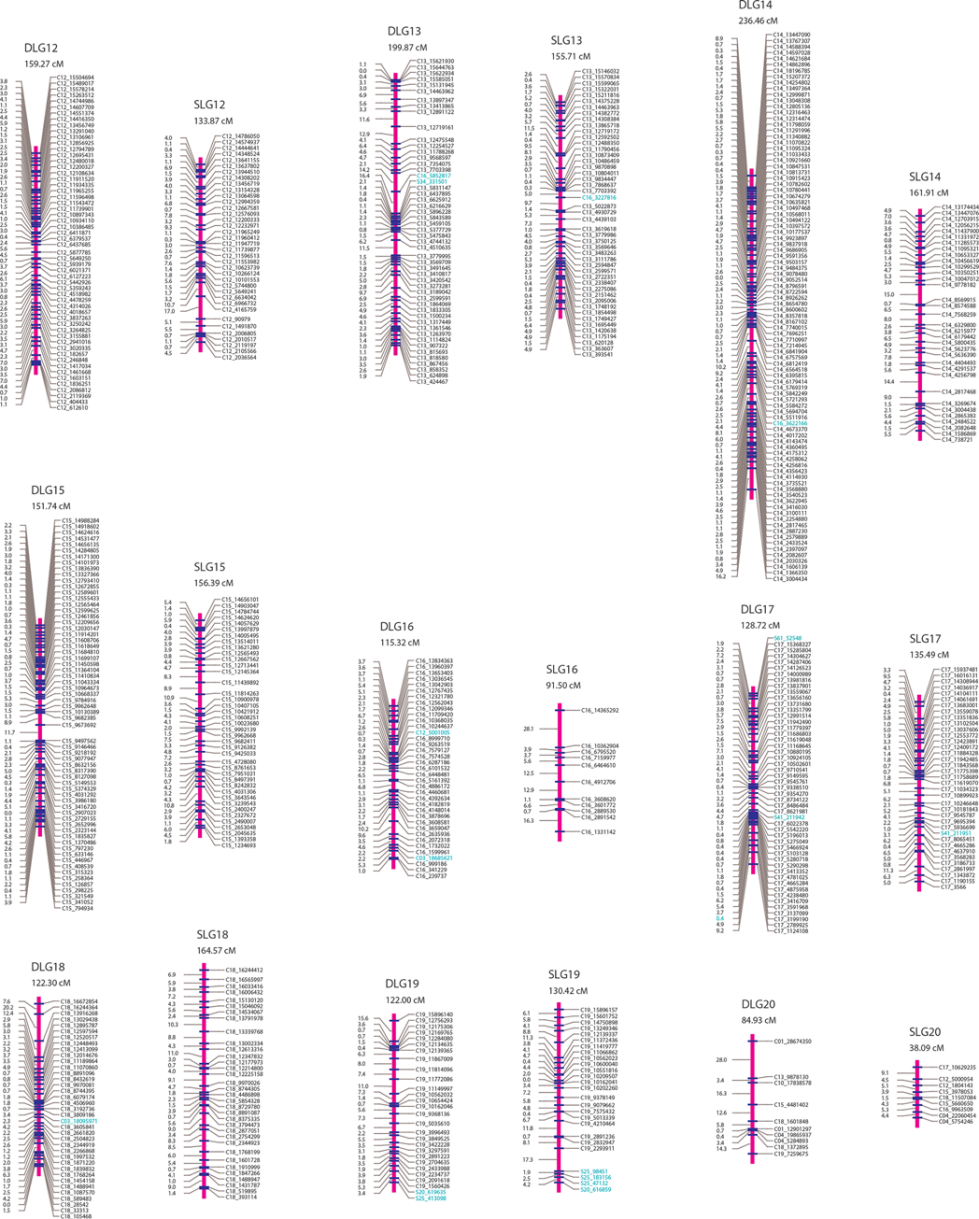
The genetic maps of linkage groups DLG12-DLG20 for the maternal *P*. *deltoides* ‘I-69’ and SLG12-SLG20 for the paternal *P*. *simonii* ‘L-3’. The length of each linkage group is given below the linkage group name. SNP marker names are denoted by the names and positions of chromosomes or scaffolds of the reference genome *P*. *trichocarpa*. Markers from other chromosomes and additional scaffolds are in green.

Two parent-specific linkage maps were constructed each with SNP markers that segregate in the ratio of 1:1 using the mapping strategy described in “Materials and Methods” (Figs 1–3). A total of 1,601 SNPs with the segregation type of *abaa* were assigned to 20 linkage groups (denoted as DLG1-20) at the LOD threshold of 15.0 and formed the genetic linkage map of the female *P*. *deltoides* ‘I-69’. On this maternal map, the lengths of linkage groups ranged from 84.93 to 505.15 cM, amounting to the total length of 4249.12 cM. On average, the distance between adjacent mapped markers was 2.69 cM, ranging from 0.00 to 27.97 cM. For the male *P*. *simonii* ‘L-3’, the linkage map was constructed with 940 SNP markers of segregation type *aaab*, which were also grouped into 20 linkage groups (denoted as SLG1-20) at the LOD threshold of 15.0. The total length of this parental map was 3816.24 cM, with an average length of 4.15 cM for marker intervals ranging from 0.00 to 28.07 cM and 190.81 cM for linkage groups ranging from 38.09 to 437.58 cM.

More detailed information on the two parental linkage maps is listed in Table 3 and S3 and S4 Tables. Except for DLG20 and SLG20, each linkage group of the two maps corresponds to the reference chromosome of *P*. *trichocarpa*. As we expected, there remained strong positive correlations among the SNP number, the genetic distance, and the physical size (Table 4). The number of SNP markers was significantly correlated with the length for each linkage group, with a high correlation coefficient of 0.9364 for the female map and 0.9376 for the male map. The correlations between the genetic and physical size for the two parents were also relatively high both at coefficients of over 0.90. Furthermore, the relationship between the SNP number and chromosome size was consistent for the two linkage maps, both with an absolutely high but relatively low correlation coefficient of ~0.85. In addition to these high consensuses for the genetic maps, we also predicted linkage phases between any two adjacent SNP markers, which were listed in the fifth column of S3 and S4 Tables. The predicted linkage phases would provide valuable information for deriving conditional probabilities of QTL genotypes on marker genotypes when performing QTL mapping in the hybrid population of *P*. *deltoides* × *P*. *simonii*.

**Table 3:** SNP number and length of linkage groups in two parental genetic maps of *P*. *deltoides* ‘I-69’ and *P*. *simonii* ‘L-3’.

*P*. *deltoides* ‘I-69’			*P*. *simonii* ‘L-3’			Chromosome size (Mb)*^c^*
Group*^a^*	SNP number	Length (cM)	Group*^b^*	SNP number	Length (cM)	
DLG1	206	505.15	SLG1	100	437.58	50.50
DLG2	125	329.32	SLG2	70	273.33	25.26
DLG3	103	259.12	SLG3	55	229.09	21.82
DLG4	87	228.02	SLG4	63	172.84	24.27
DLG5	99	254.53	SLG5	70	260.96	25.89
DLG6	120	354.98	SLG6	79	292.60	27.91
DLG7	58	160.01	SLG7	36	161.42	15.61
DLG8	109	220.67	SLG8	57	225.95	19.47
DLG9	90	210.65	SLG9	44	171.96	12.95
DLG10	126	221.09	SLG10	66	248.79	22.58
DLG11	53	184.97	SLG11	25	137.77	18.50
DLG12	56	159.27	SLG12	37	133.87	15.76
DLG13	51	199.87	SLG13	47	155.71	16.32
DLG14	92	236.46	SLG14	34	161.91	18.92
DLG15	64	151.74	SLG15	40	156.39	15.28
DLG16	37	115.32	SLG16	11	91.50	14.49
DLG17	50	128.72	SLG17	37	135.49	16.08
DLG18	37	122.30	SLG18	34	164.57	16.96
DLG19	28	122.00	SLG19	26	130.42	15.94
DLG20	10	84.93	SLG20	9	38.09	
Total	1601	4249.12		940	3816.24	394.51

^*a*^DLG indicates the linkage group of *P*. *deltoides* 'I-69';

^*b*^SLG indicates the linkage group of *P*. *simonii* 'L-3';

^*c*^The genome size refers to the reference genome of *P*. *trichocarpa* (Tuskan et al. 2006).

**Table 4:** Correlations among the SNP number, genetic length and chromosome size for the linkage groups of the two parental maps.

	SNP number in DLG	DLG Length	SNP number in SLG	SLG length
DLG Length	0.9364			
SNP number in SLG	0.9010	0.8905		
SLG length	0.9421	0.9394	0.9376	
Chromosome size	0.8673	0.9149	0.8437	0.9216

To demonstrate the potential applications of the linkage maps constructed above in QTL mapping, we preliminarily performed composite interval mapping of the tree height and DBH. S1–S4 Figs showed the profiles of LRs against the map positions and the thresholds for existing QTLs determined by 1000 permutation tests. As a result, 8 QTLs for tree height and 7 for DBH were significantly identified and they explained 81.1% and 70.4% of the phenotypic variances, respectively (S5 and S6 Tables).

### Synteny and Collinearity between Genetic and Physical Maps

**Fig 4:**
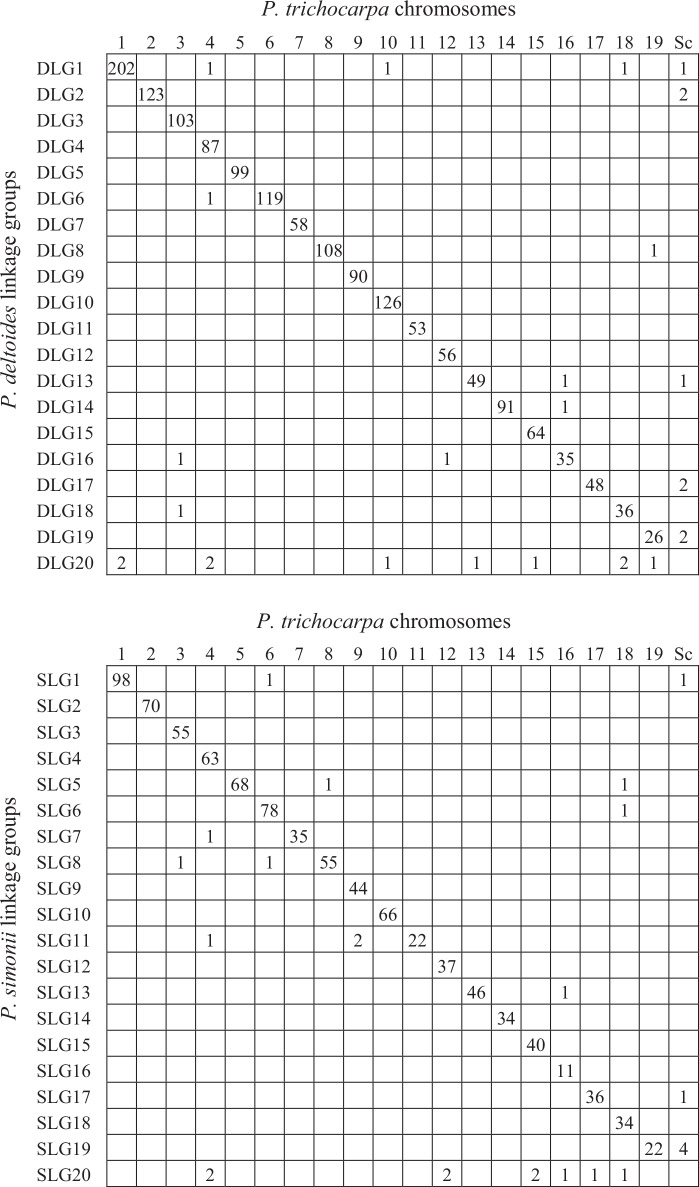
Oxford grid comparison of the genetic and physical maps. Each number in a cell denotes the number of homologous pair of SNP markers in each linkage group or genome. The last columns correspond to all the additional scaffolds of *P*. *trichocarpa*.

Oxford grid comparison of the genetic and physical maps showed that high levels of synteny were conserved between the parental genomes and the reference genome of *P*. *trichocarpa* (Fig 4). It can be found that all the SNPs on each of the 9 female linkage groups (DLGs 3, 4, 5, 7, 9, 10, 11, 12, 15) and 10 male linkage groups (SLGs 2, 3, 4, 9, 10, 12, 14, 15, 16, 18) were located in the corresponding chromosomes of *P*. *trichocarpa* (Fig 4). For the other linkage groups, except for DLG 20 and SLG 20, only a few (1–4) SNPs were identified in discordant reference chromosomes. However, synteny between DLG 20 (or SLG 20) and the physical map was inconclusive because all the 10 (9) SNPs were found in 7 (6) different reference chromosomes. Of all the 1,601 SNPs on the female genetic map of *P*. *deltoides*, 98.3% had a one-to-one relationship between the genetic linkage groups and the reference chromosomes, indicating highly conserved synteny between *P*. *deltoides* and *P*. *trichocarpa*. A similar high level of synteny between *P*. *simonii* and *P*. *trichocarpa* was also retained because 97.2% SNP markers on the male genetic map had a one-to-one relationship with the physical map.

**Fig 5:**
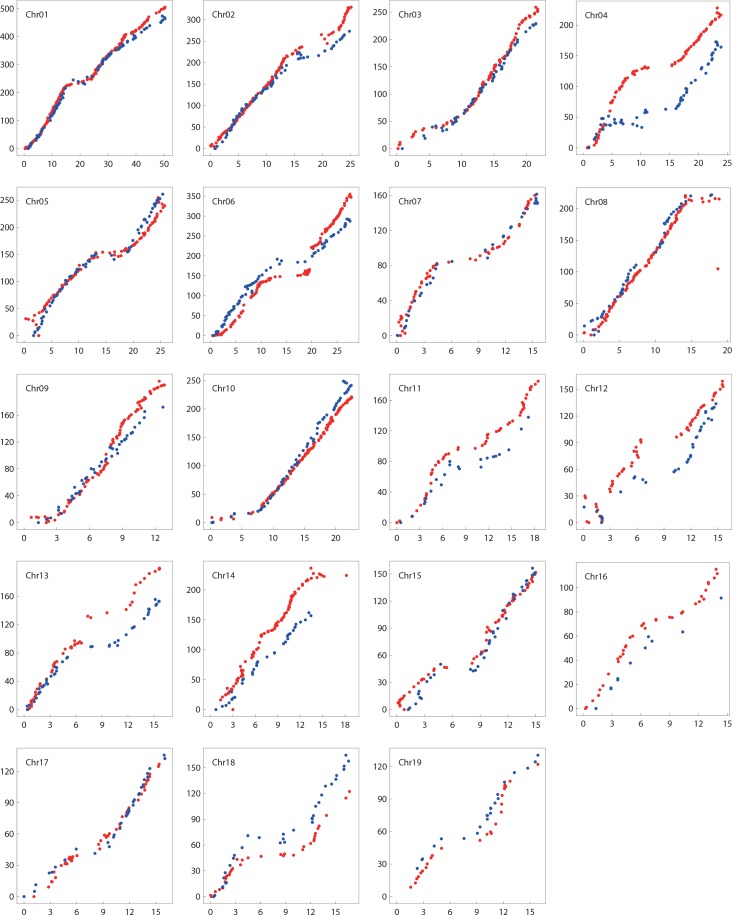
Collinear comparison of the genetic and physical maps. The x-axis indicates the reference sequence position with the unit of Mbp; the y-axis indicates the genetic map position with the unit of cM. The red and blue points, respectively, indicate the SNP position on the female and male genetic maps against the reference genome position.

Further comparison of the linkage groups with the physical map revealed extensive conserved marker order except for a few of inversions in each chromosome (Fig 5). All the 19 syntenic pairs of linkage groups and chromosomes showed apparent collinearity between the female parent *P*. *deltoides* and *P*. *trichocarpa*, and between the male parent *P*. *simonii* and *P*. *trichocarpa*. However, there existed some linkage groups exhibiting rearrangements in local regions. For DLGs 2, 4, 8, 14, 15 and SLGs 2, 4, 6, 10, 11, 12, 17, a few short dot lines in Fig 5 were perpendicular to the 45-degree line, suggesting inverse orders of some SNPs relative to the reference genome positions.

## Discussion

Here, we reported the high-quality SNP linkage maps of *P*. *deltoides* and *P*. *simonii* with the most recent RAD sequencing technology. The two parent-specific linkage maps will serve as important genetic resources for identifying QTLs that contribute to growth and timber traits as well as resistance with different QTL mapping approaches implemented in such an F_1_ hybrid population of *P*. *deltoides* and *P*. *simonii* [46,47]. The two linkage maps will also allow for comparative genomics among different species of *Populus*, and provide additional information on evolution in poplar. The less number of SNPs on the male linkage map suggests that the male parent *P*. *simonii* ‘L-3’ is less heterozygous than the female parent *P*. *deltoides* ‘I-69’. Each parental linkage map with a total of 20 linkage groups closely matches the karyotype of *Populus* (2n = 38). The last group DLG20 of the female map would be merged into group DLG4 with a large interval distance of 71.4 cM if the LOD threshold for linkage grouping decreased to a lower value of 8.0. Similarly, group SLD20 of the male map would be incorporated into group SLD8 with a large interval distance of 39.8 cM if the LOD threshold were chosen as 6.0. These suggested that the last small linkage group of each parental map might collapse with another linkage group when additional markers are provided. Although a few SNP markers come from other chromosomes and there exist a few apparent local discrepancies in marker order on some linkage groups (Figs 4 and 5), there are no apparent rearrangements and thus highly conserved synteny and collinearity can be inferred between the parental maps and the reference genome. In addition, on the parental maps there are 14 SNPs from the additional scaffolds of the reference genome, which could link unmapped scaffolds 47, 36, 34, 41, and 20 to chromosomes 1, 2, 13, 17, and 19 in poplar, respectively (Fig 4, S7 Table). Apparently, the result of synteny and collinearity was based on SNPs from conserved genome regions of different *Populus* species. However, there would likely be much less synteny and collinearity if divergent regions of the genomes were considered.

It is desirable to construct an integrated linkage map in an F_1_ population generated by hybridizing two individual trees [11, 21]. However, like most linkage mapping studies in forest trees [7, 12, 17], we had to build two linkage maps each specially for one parent since there were no enough markers heterozygous in both parents with segregation type *abab* or *abcd* as bridges, and any two markers one with segregation type *abaa* and the other *aaab* cannot provide any linkage information. The main reason why the overwhelming number of SNPs were not selected in our linkage analysis is that many individuals were not genotyped at most SNPs owning to low or no read coverage (S5 Fig). Another reason is that there were a lot of distorted SNPs not following Mendelian segregation ratio (*p* < 0.05), which accounted for 41.3% of the SNPs with segregation type *abaa* and 47.3% *aaab* that each has at least 50 individuals genotyped (S6 Fig). Segregation distortion was previously thought to be attributed to factors such as chromosome loss, genetic isolating mechanisms, viability genes and even genotyping errors [48], but one recent NGS study revealed that gene conversion events were unexpectedly abundant during meiosis in *Arabidopsis* [49], which skew segregation rates of alleles and were typically ignored in linkage mapping. Of all the SNPs genotyped in both parents, 77.9% (300,107) were homozygous and could not segregate in the progeny (Table 2), which suggested that a considerable number of SNP markers would segregate in a ratio of 1:2:1 in an F_2_ hybrid population.

The two parental maps may be in an excess of genetic map size compared with most previous poplar maps. Since obtaining the true order of a large number of markers in a linkage group is challenging, we chose the optimal orders based on SARF among several ordering results from JoinMap and FsLinkageMap (see Materials and Methods). We used the ML method in JoinMap because it can build a more accurate genetic map in a full-sib family of an outbreeding species than the previous regression method [50]. The genetic distances between adjacent markers were directly calculated from the results of two-point linkage analysis. This could expand the size of linkage maps because the double recombinants were not considered properly, but the more accurate marker orders and genetic distances between adjacent markers are most valuable in comparative genomics and in identifying QTLs [44,51]. The total sizes of our two linkage maps matched the results with the ML mapping algorithm in JoinMap 4.1, but disagreed substantially with the results from the regression algorithm in the same software (S8 Table). Interestingly, the results of the regression algorithm almost matched the estimates using the method of Hulbert et al. [52], which resulted in 2,085.83 cM for the female map and 2,434.59 cM for the male map. Investigations into the previous studies of genetic mapping in poplar showed that the observed genome length ranged from 1,600 to 3,800 cM [16, 53]. These discrepancies between different studies or between different algorithms even with the same software and data may reflect the difficulty of obtaining a perfect linkage map with high-density and high-quality in *Populus*.

## Conclusions

We have constructed high-density genetic linkage maps of the maternal *Populus deltoides* ‘I-69’ and paternal *P*. *simonii* ‘L3’ in the F_1_ hybrid population. We demonstrate that RAD sequencing technology is capable of generating a large number of SNPs in a fast and cost-effective way for constructing genetic maps in outbred forest trees. Further analysis reveals that the synteny and collinearity are highly conserved between the parental linkage maps and the reference genome of *P*. *trichocarpa*. The linkage maps provide useful genetic resources for detecting QTLs that control growth and timber traits, especially that underlie the developmental trajectories in the permanent *Populus* population.

## Supporting Information

S1 Fig The profile of the log-likelihood ratios (LR) of detecting QTLs for tree height based on the composite interval mapping method and the linkage map of *P*. *deltoides* ‘I-69’. The threshold value for asserting the existence of a QTL at the significant level p = 0.05 is indicated as horizontal dashed lines, which was determined by 1000 permutation tests.
(PDF)

S2 Fig The profile of the log-likelihood ratios (LR) of detecting QTLs for diameter at breast height (DBH) based on the composite interval mapping method and the linkage map of *P*. *deltoides* ‘I-69’. The threshold value for asserting the existence of a QTL at the significant level p = 0.05 is indicated as horizontal dashed lines, which was determined by 1000 permutation tests.
(PDF)

S3 Fig The profile of the log-likelihood ratios (LR) of detecting QTLs for tree height based on the composite interval mapping method and the linkage map of *P*. *simonii* ‘L-3’. The threshold value for asserting the existence of a QTL at the significant level p = 0.05 is indicated as horizontal dashed lines, which was determined by 1000 permutation tests.
(PDF)

S4 Fig The profile of the log-likelihood ratios (LR) of detecting QTLs for diameter at breast height (DBH) based on the composite interval mapping method and the linkage map of *P*. *simonii* ‘L-3’. The threshold value for asserting the existence of a QTL at the significant level p = 0.05 is indicated as horizontal dashed lines, which was determined by 1000 permutation tests.
(PDF)

S5 Fig Boxplots of the numbers of individuals genotyped at SNPs for segregation types *abaa* and *aaab*. The median and 75% quantile are 54 and 135 for *abaa*, and 54 and 134 for *aaab*.
(PDF)

S6 Fig Boxplots of p-values corresponding to chi-square tests if each SNP segregates in a ratio of 1:1 for the two segregation types. Those SNPs with less than 50 individuals genotyped were excluded. The Medians are 0.1317 and 0.0699 for *abaa* and *aaab*. The *p*-value of 0.05 corresponds to 41.3% quantile for *abaa* and 47.3% for *aaab*.
(PDF)

S1 Table Average coverage depth and standard deviation of the unique mapped HQ RADSeq reads and their coverage of the reference genome for each of the parents and progeny. (XLSX)

S2 Table Number of SNP loci both genotyped with two data sets from NBI and BGI, and confirmed with each other, for the 2545 mapping SNPs. (DOCX)

S3 Table Detailed information on genetic distance and linkage phase between adjacent SNP markers on the genetic linkage map of the female *P*. *deltoides* ‘I-69’. (XLSX)

S4 Table Detailed information on genetic distance and linkage phase between adjacent SNP markers on the genetic linkage map of the male *P*. *simonii* ‘L-3’. (XLSX)

S5 Table Detected QTLs for tree height and diameter at breast height (DBH) based on the female linkage map of *P*. *deltoides* ‘I-69’ and the composite interval mapping method. (DOCX)

S6 Table Detected QTLs for tree height and diameter at breast height (DBH) based on the female linkage map of *P*. *simonii* ‘L-3’ and the composite interval mapping method. (DOCX)

S7 Table 14 SNPs from additional scaffolds of the reference genome, *P*. *trichocarpa*. (DOCX)

S8 Table Linkage group lengths (cM) with different mapping methods for the parental genetic maps of *P*. *deltoides* and *P*. *simonii*. (DOCX)
