# Supplementary figures and images for: Construction of High-Density Linkage Maps of Populus deltoides × P. simonii Using Restriction-Site Associated DNA Sequencing
Source: PLoS One. 2016 Mar 10;11(3):e0150692. doi: 10.1371/journal.pone.0150692 (PMC4786213; doi:10.1371/journal.pone.0150692)

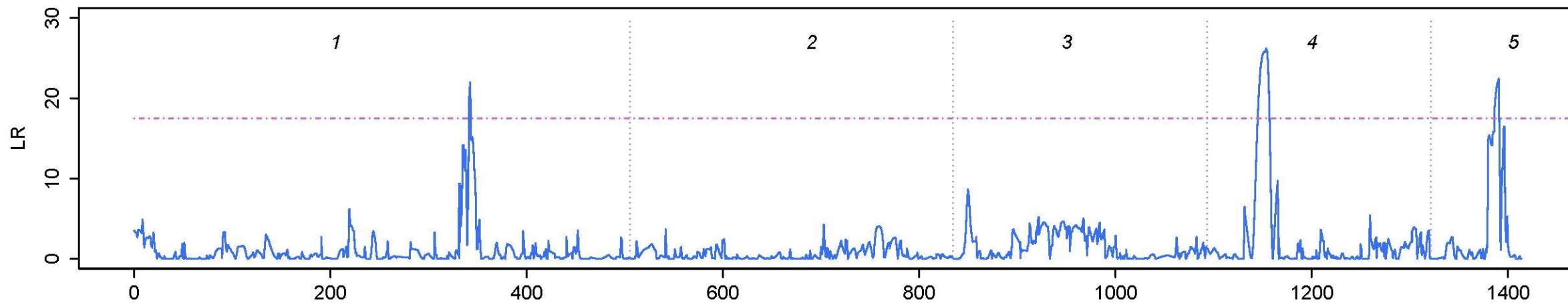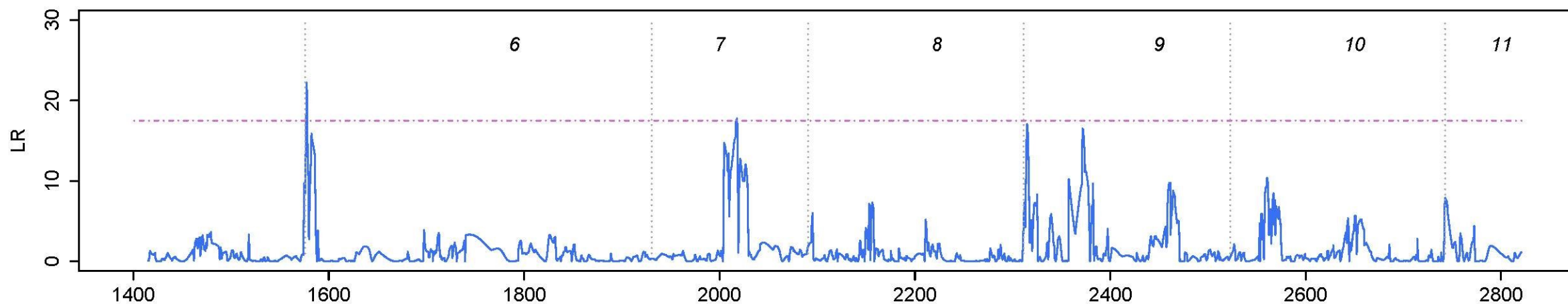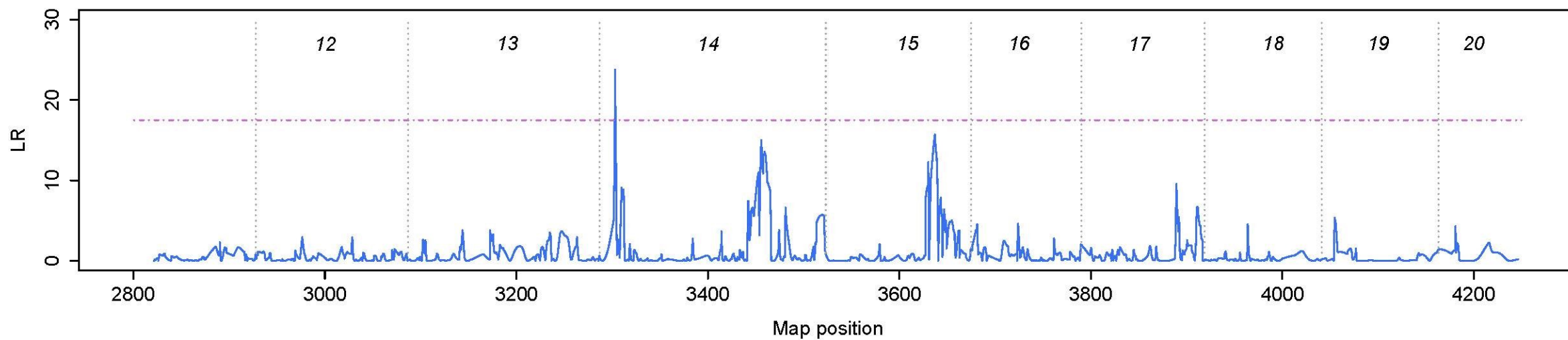

Supplement: S1 Fig — The threshold value for asserting the existence of a QTL at the significant level p = 0.05 is indicated as horizontal dashed lines, which was determined by 1000 permutation tests. (PDF) [file pone.0150692.s001.pdf]

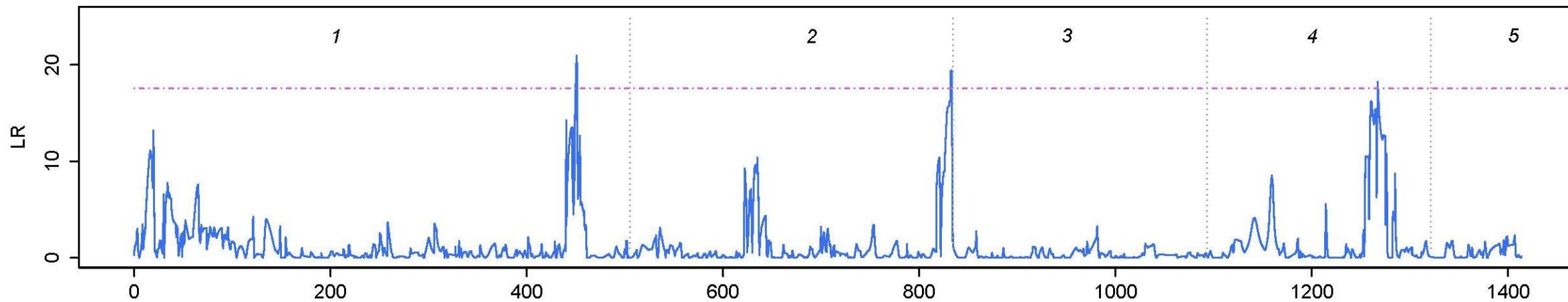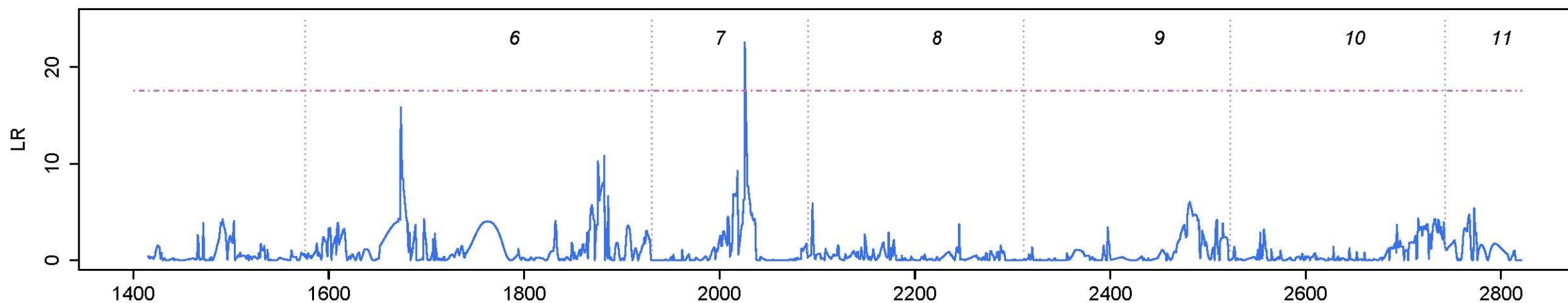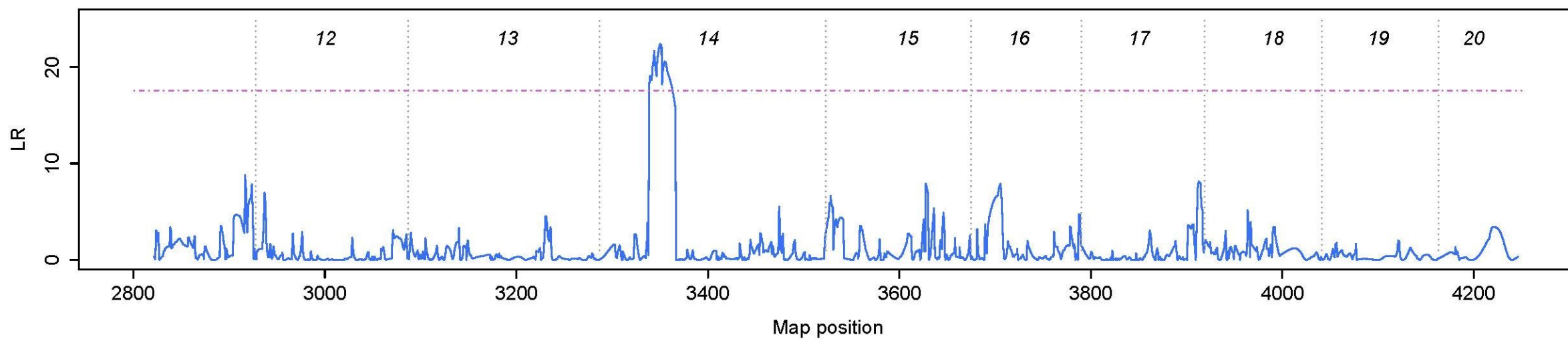

Supplement: S2 Fig — The threshold value for asserting the existence of a QTL at the significant level p = 0.05 is indicated as horizontal dashed lines, which was determined by 1000 permutation tests. (PDF) [file pone.0150692.s002.pdf]

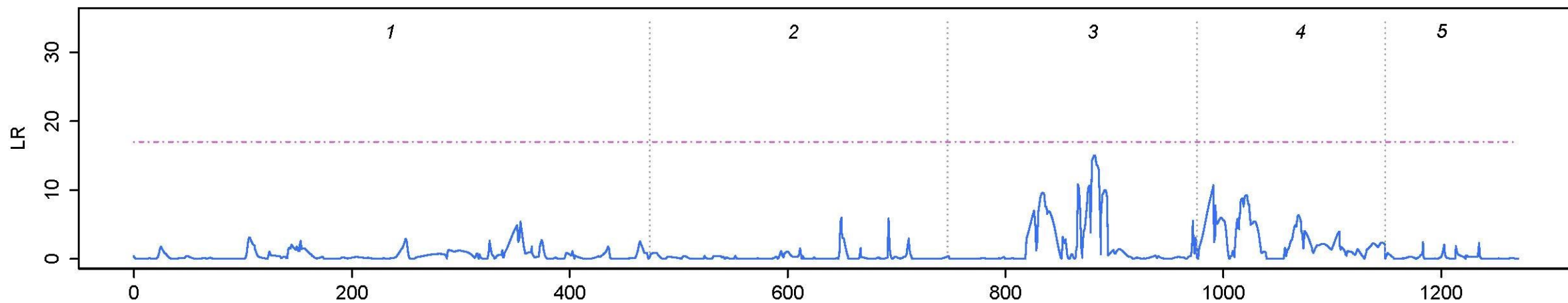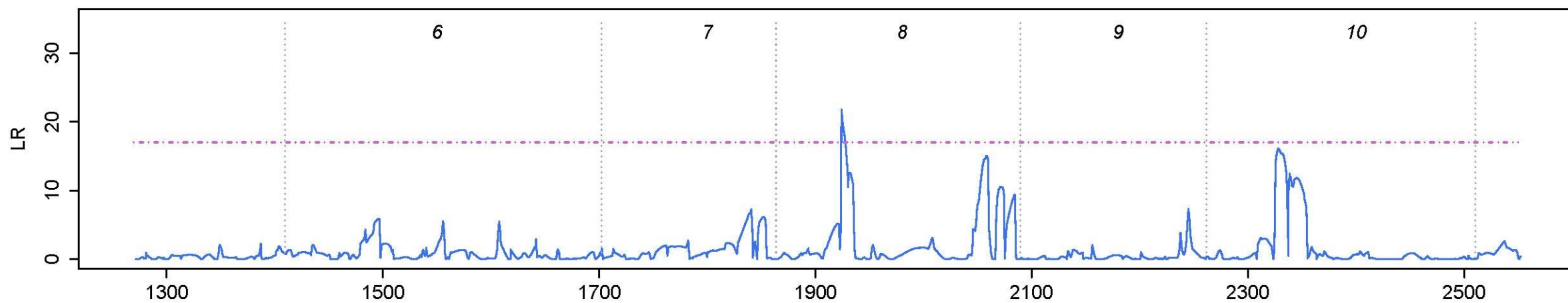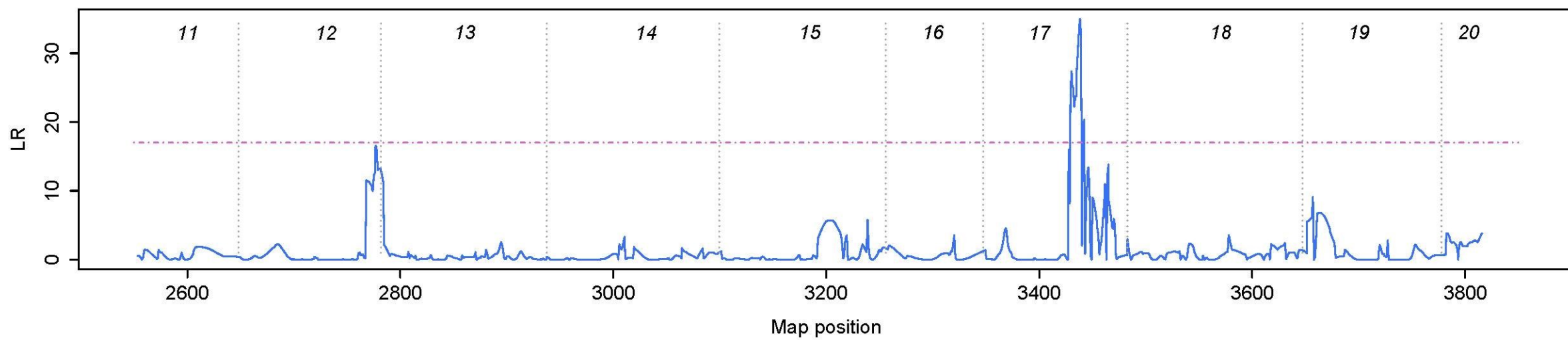

Supplement: S3 Fig — The threshold value for asserting the existence of a QTL at the significant level p = 0.05 is indicated as horizontal dashed lines, which was determined by 1000 permutation tests. (PDF) [file pone.0150692.s003.pdf]

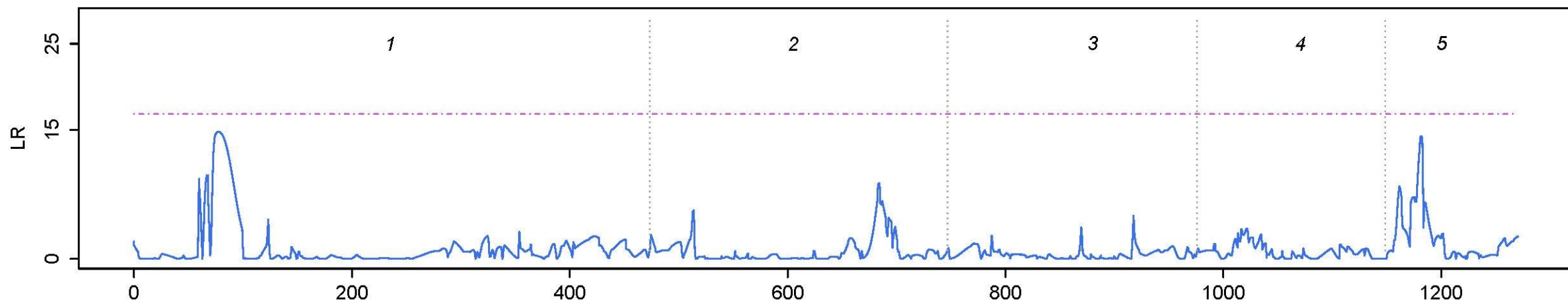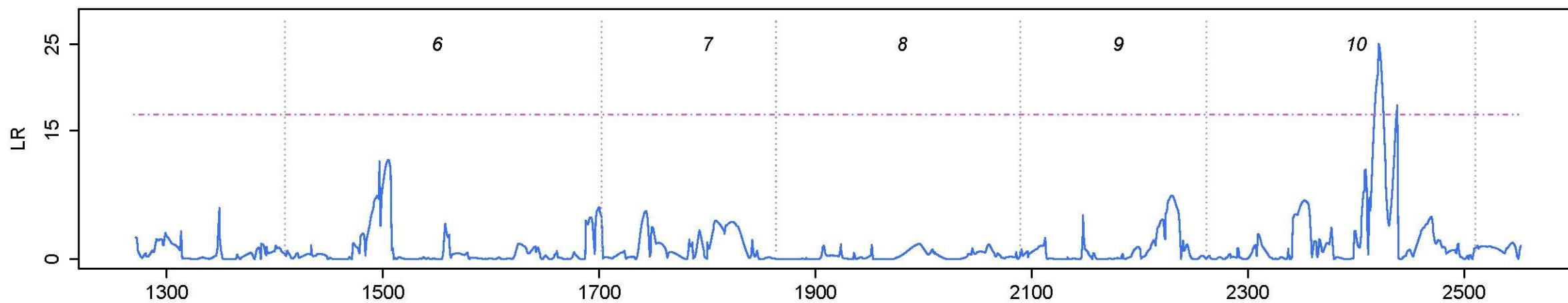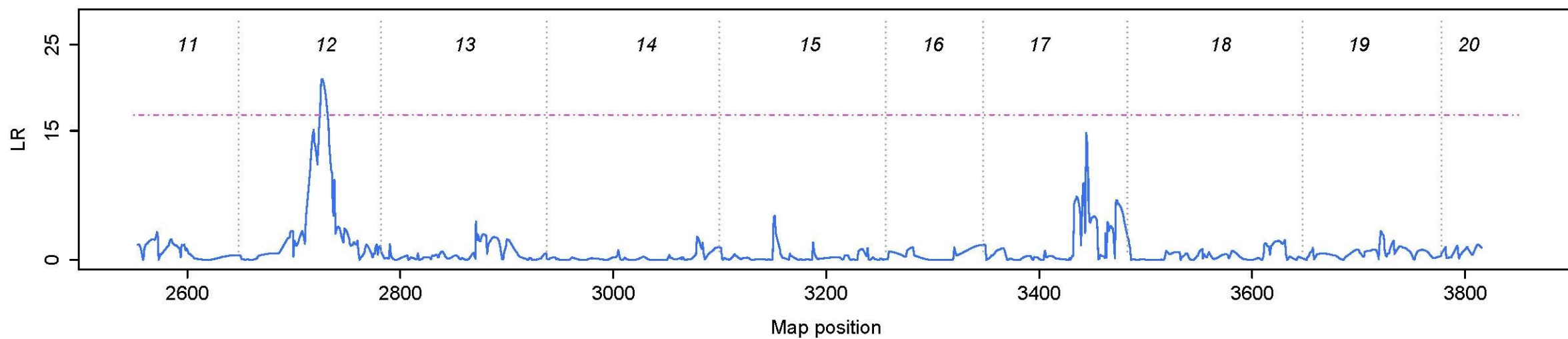

Supplement: S4 Fig — The threshold value for asserting the existence of a QTL at the significant level p = 0.05 is indicated as horizontal dashed lines, which was determined by 1000 permutation tests. (PDF) [file pone.0150692.s004.pdf]

Number of individuals

300  
250  
200  
150  
100  
50  
0

abxaa

aaxab

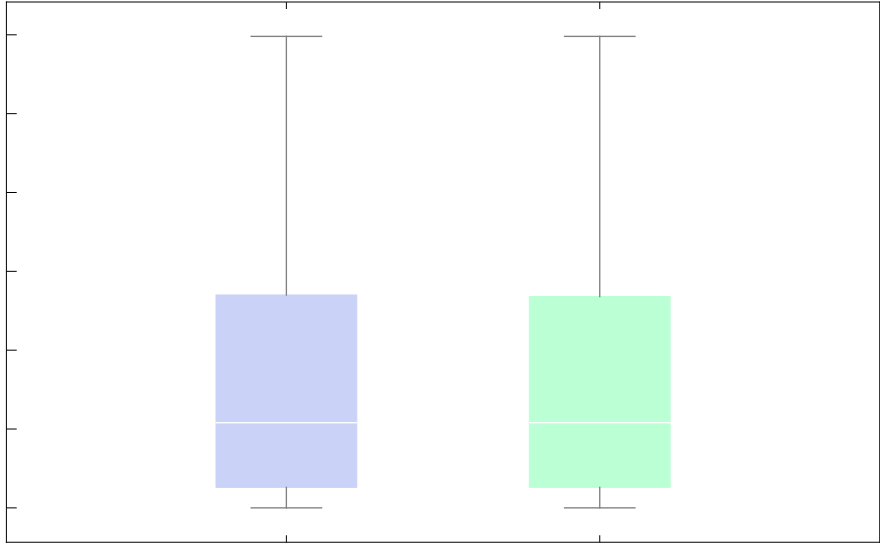

Supplement: S5 Fig — The median and 75% quantile are 54 and 135 for abaa, and 54 and 134 for aaab. (PDF) [file pone.0150692.s005.pdf]

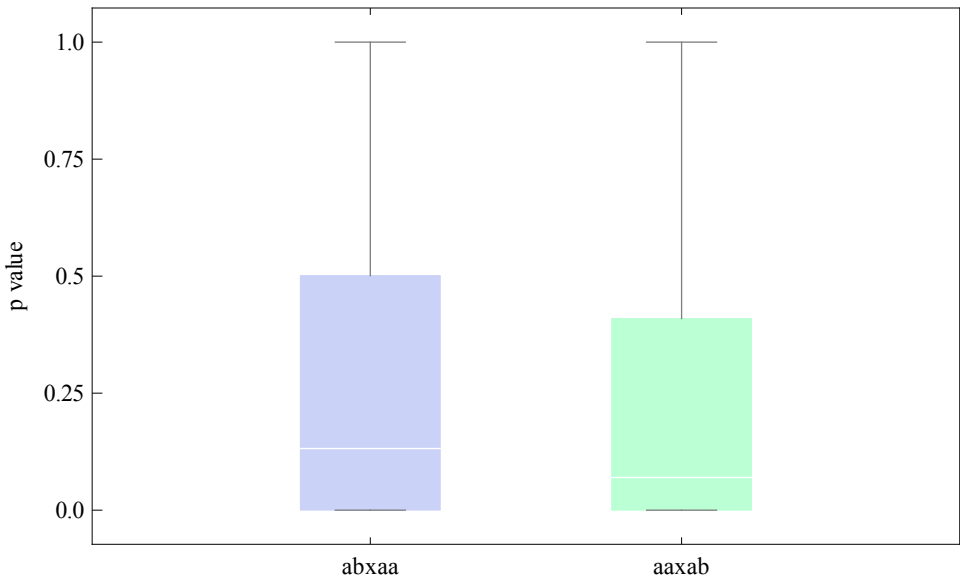

Supplement: S6 Fig — Those SNPs with less than 50 individuals genotyped were excluded. The Medians are 0.1317 and 0.0699 for abaa and aaab. The p-value of 0.05 corresponds to 41.3% quantile for abaa and 47.3% for aaab. (PDF) [file pone.0150692.s006.pdf]
