# Supplementary material for: Construction of High-Density Linkage Maps of Populus deltoides × P. simonii Using Restriction-Site Associated DNA Sequencing
Source: PLoS One. 2016 Mar 10;11(3):e0150692. doi: 10.1371/journal.pone.0150692 (PMC4786213; doi:10.1371/journal.pone.0150692)
Supplement: S2 Table — (DOCX) [file pone.0150692.s008.docx]

**S2 Table.** **Number of SNP loci both genotyped with two data sets from NBI and BGI, and confirmed with each other, for the 2545 mapping SNPs**

| Sample | Loci both genotyped  with two data sets | SNP genotype confirmed  with two data sets | Ratio |
| --- | --- | --- | --- |
| Female parent ‘I-69’ | 2537 | 2479 | 0.9771 |
| Male parent ‘L-3’ | 2476 | 2440 | 0.9855 |
| Progeny ‘C15-1’ | 2483 | 2442 | 0.9835 |
| Total | 7496 | 7361 | 0.9820 |

**S1 Fig. 1.** **Boxplots of the numbers of individuals genotyped at SNPs for segregation types *abaa* and *aaab*.** The median and 75% quantile are 54 and 135 for *abaa*, and 54 and 134 for *aaab*.

**S1 Fig. 2.**  **Boxplots of p-values corresponding to chi-square tests if each SNP segregates in a ratio of 1:1 for the two segregation types.** Those SNPs with less than 50 individuals genotyped were excluded. The Medians are 0.1317 and 0.0699 for *abaa* and *aaab*. The *p*-value of 0.05 corresponds to 41.3% quantile for *abaa* and 47.3% for *aaab*.

| 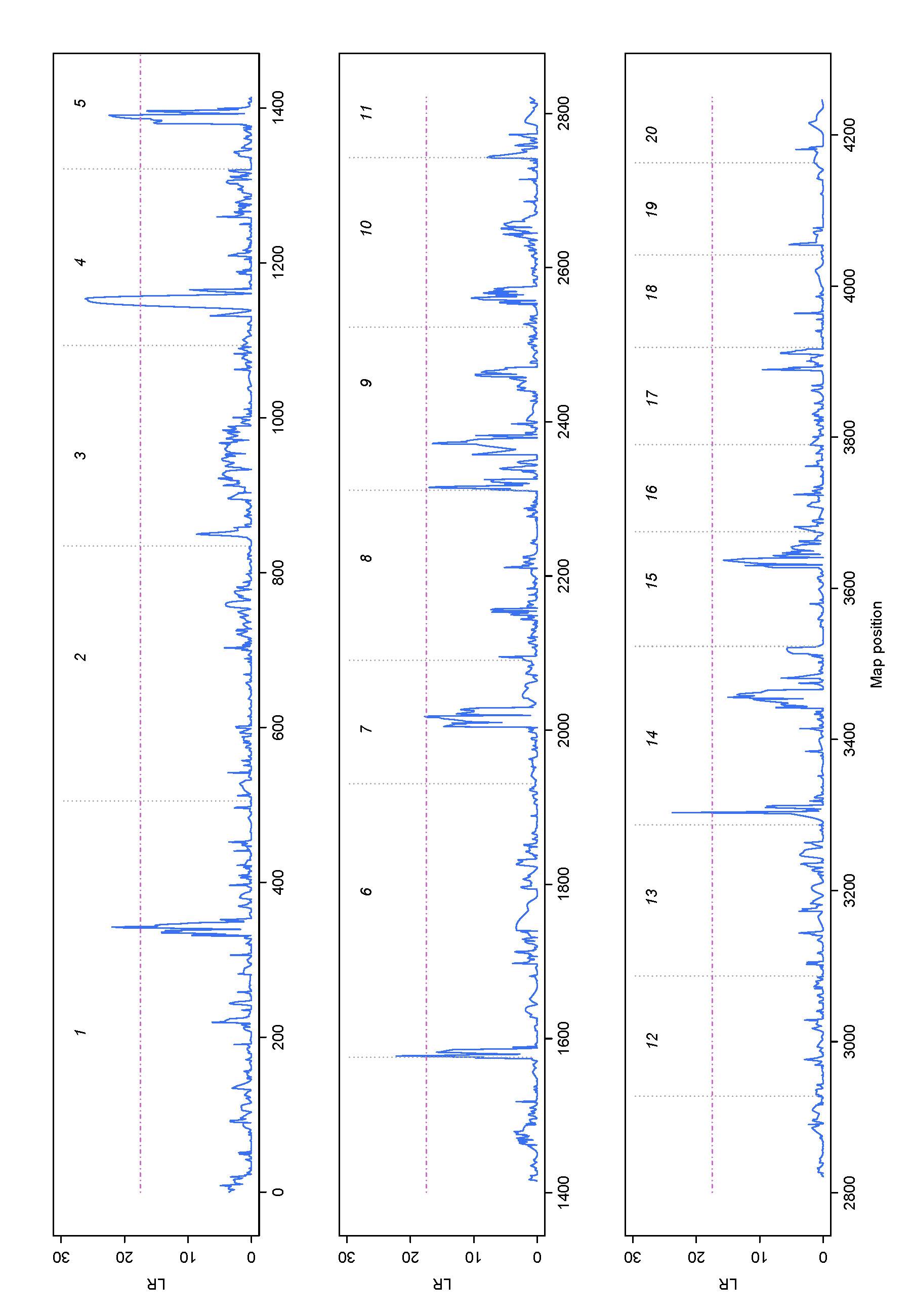 | **S1 Fig. 3.** **The profile of the log-likelihood ratios (LR) of detecting QTLs for tree height based on the composite interval mapping method and the linkage map of *P. deltoides* ‘I-69’**. The threshold value for asserting the existence of a QTL at the significant level p=0.05 is indicated as horizontal dashed lines, which was determined by 1000 permutation tests. |
| --- | --- |

| 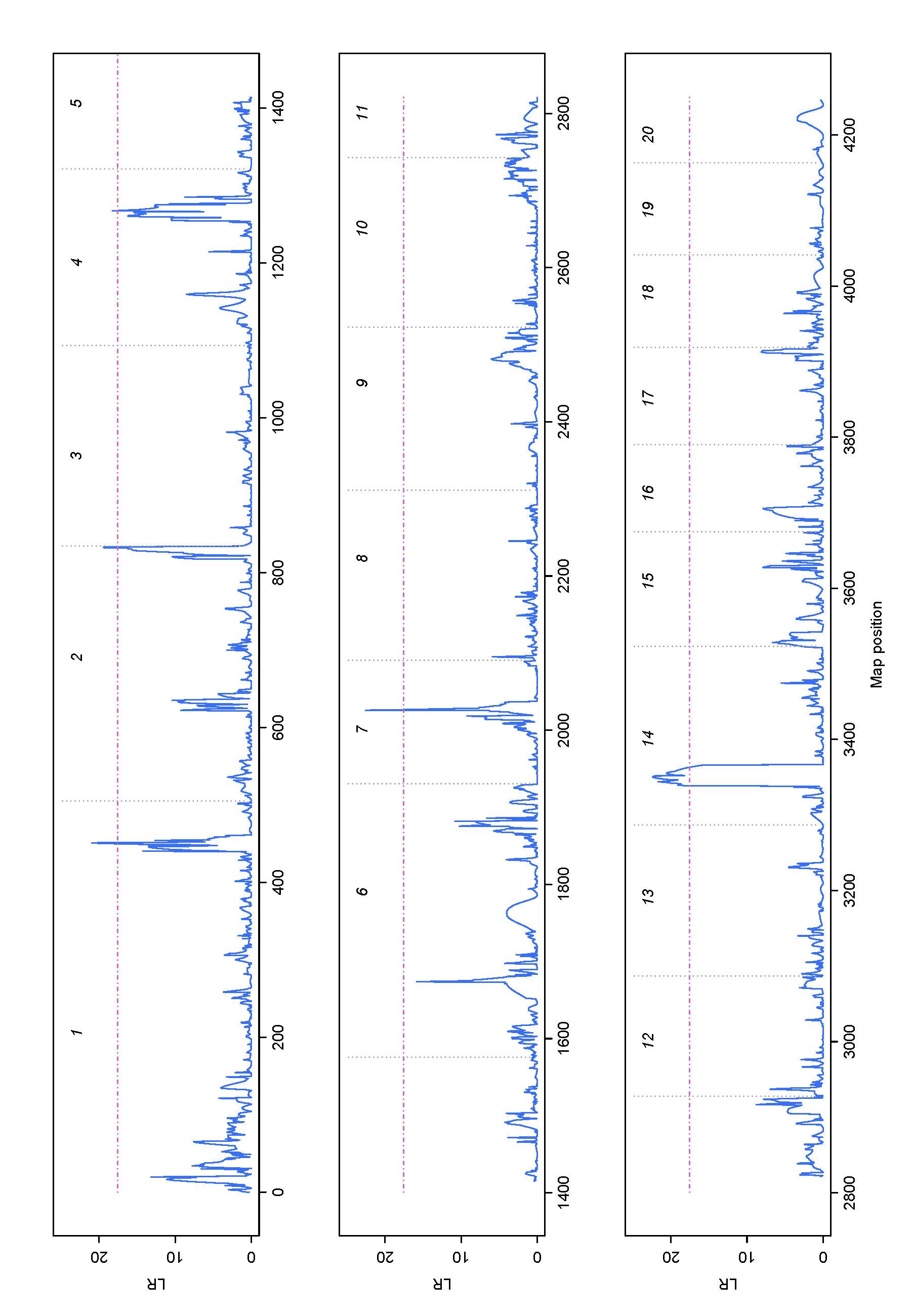 | **S1 Fig. 4.** **The profile of the log-likelihood ratios (LR) of detecting QTLs for diameter at breast height (DBH) based on the composite interval mapping method and the linkage map of *P. deltoides* ‘I-69’**. The threshold value for asserting the existence of a QTL at the significant level p=0.05 is indicated as horizontal dashed lines, which was determined by 1000 permutation tests. |
| --- | --- |

| 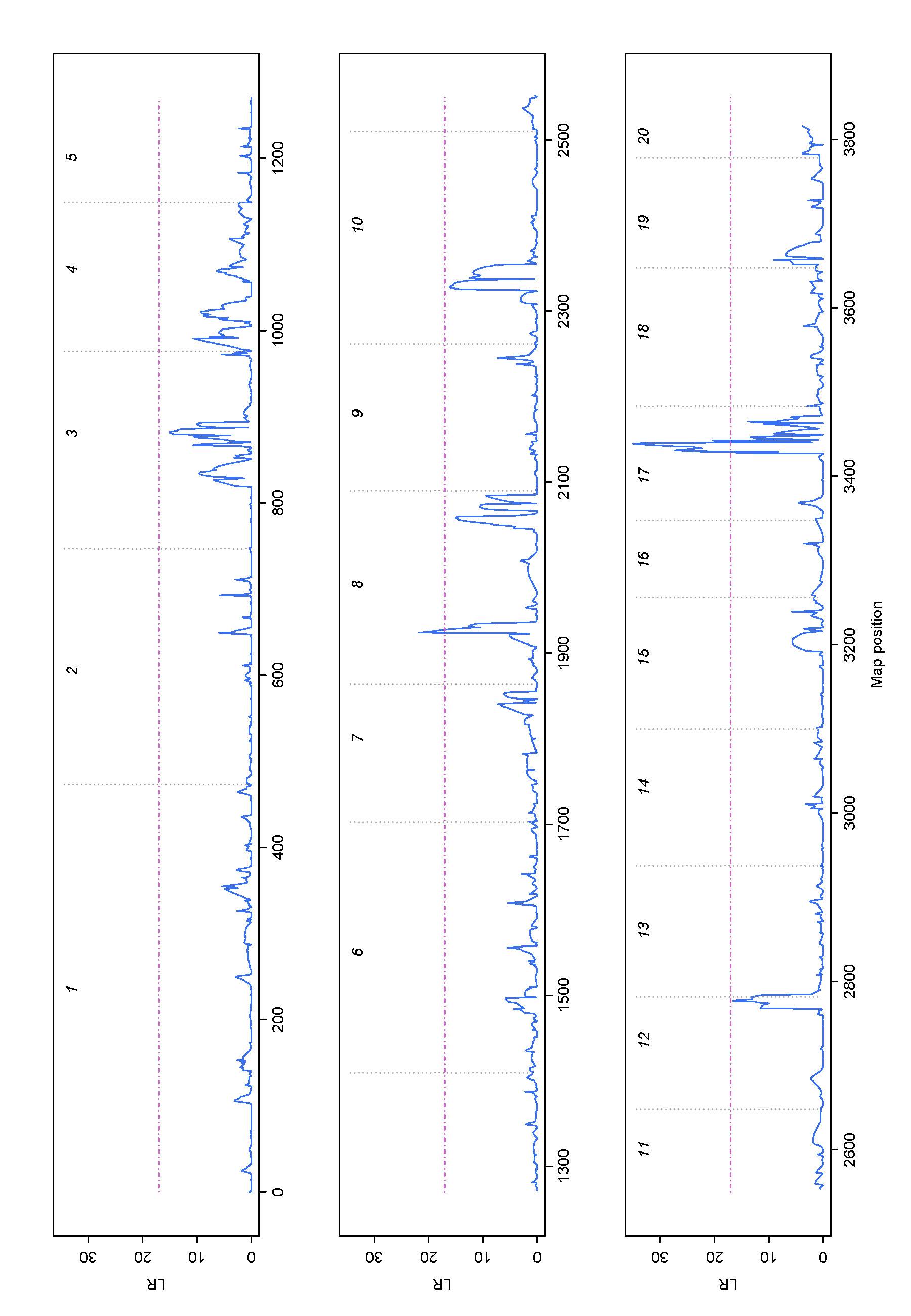 | **S1 Fig. 5.** **The profile of the log-likelihood ratios (LR) of detecting QTLs for tree height based on the composite interval mapping method and the linkage map of *P. simonii* ‘L-3’**. The threshold value for asserting the existence of a QTL at the significant level p=0.05 is indicated as horizontal dashed lines, which was determined by 1000 permutation tests. |
| --- | --- |
| 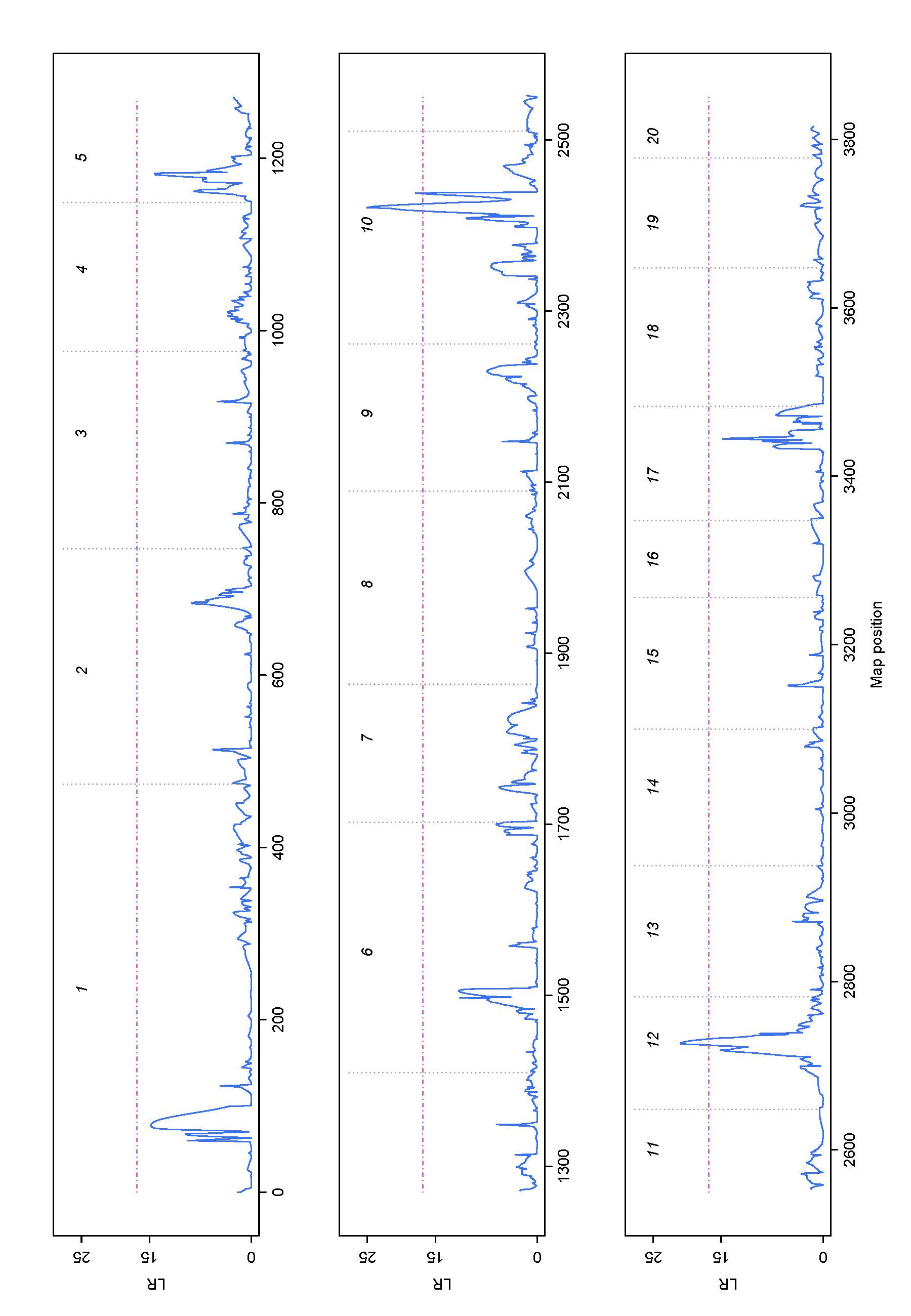 | **S1 Fig. 6.** **The profile of the log-likelihood ratios (LR) of detecting QTLs for diameter at breast height (DBH) based on the composite interval mapping method and the linkage map of *P.* *simonii* ‘L-3’**. The threshold value for asserting the existence of a QTL at the significant level p=0.05 is indicated as horizontal dashed lines, which was determined by 1000 permutation tests. |
