# Supplementary material for: Construction of High-Density Linkage Maps of Populus deltoides × P. simonii Using Restriction-Site Associated DNA Sequencing
Source: PLoS One. 2016 Mar 10;11(3):e0150692. doi: 10.1371/journal.pone.0150692 (PMC4786213; doi:10.1371/journal.pone.0150692)
Supplement: S5 Table — (DOCX) [file pone.0150692.s011.docx]

**S5 Table. Detected QTLs for tree height and diameter at breast height (DBH) based on the female linkage map of *P. deltoides* ‘I-69’ and the composite interval mapping method**

| Trait | QTL | Linkage  Group | Interval | Location (cM) | LR | Genetic Effect | Heritability |
| --- | --- | --- | --- | --- | --- | --- | --- |
| Tree Height | 1 | 1 | C01_30297402―C01_30968145 | 343.35 | 21.97 | 14.95 | 0.055 |
|  | 2 | 4 | C04_4641026―C04_4794671 | 61.04 | 26.19 | 17.84 | 0.079 |
|  | 3 | 5 | C05_4965586―C05_5336563 | 70.29 | 22.41 | -20.30 | 0.102 |
|  | 4 | 6 | C06_2167213―06_2117993 | 2.14 | 22.22 | 13.55 | 0.045 |
|  | 5 | 7 | C07_8088072―C07_9177987 | 87.72 | 17.77 | -11.73 | 0.034 |
|  | 6 | 14 | C14_1366350―C14_1606139 | 17.19 | 23.78 | -28.15 | 0.196 |
|  |  |  |  |  |  |  |  |
| DBH | 1 | 1 | C01_43031098―C01_43069380 | 450.31 | 20.94 | 0.19 | 0.123 |
|  | 2 | 2 | C02_24612388―C02_25139284 | 328.25 | 19.42 | 0.11 | 0.038 |
|  | 3 | 4 | C04_19737359―C04_20030489 | 174.71 | 18.26 | -0.1 | 0.034 |
|  | 4 | 7 | C07_10072659―C07_9787359 | 95.92 | 22.56 | -0.14 | 0.067 |
|  | 5 | 14 | C14_4258062―C14_4175312 | 64.28 | 22.37 | 0.12 | 0.045 |
