# Supplementary material for: Construction of High-Density Linkage Maps of Populus deltoides × P. simonii Using Restriction-Site Associated DNA Sequencing
Source: PLoS One. 2016 Mar 10;11(3):e0150692. doi: 10.1371/journal.pone.0150692 (PMC4786213; doi:10.1371/journal.pone.0150692)
Supplement: S6 Table — (DOCX) [file pone.0150692.s012.docx]

**S6 Table. Detected QTLs for tree height and diameter at breast height (DBH) based on the female linkage map of *P. simonii* ‘L-3’ and the composite interval mapping method**

| Trait | QTL | Linkage  Group | Interval | Location (cM) | LR | Genetic Effect | Heritability |
| --- | --- | --- | --- | --- | --- | --- | --- |
| Tree Height | 1 | 8 | C08_4372986―C08_4747978 | 59.08 | 21.79 | -26.45 | 0.173 |
|  | 2 | 17 | C17_12409172―C17_12423891 | 87.61 | 34.99 | -23.60 | 0.137 |
|  |  |  |  |  |  |  |  |
| DBH | 1 | 10 | C10_17034241―C10_17070204 | 156.49 | 25.06 | 0.32 | 0.339 |
|  | 2 | 12 | C12_11965249―C12_12232971 | 75.67 | 21.01 | 0.13 | 0.058 |
