# Supplementary material for: Construction of High-Density Linkage Maps of Populus deltoides × P. simonii Using Restriction-Site Associated DNA Sequencing
Source: PLoS One. 2016 Mar 10;11(3):e0150692. doi: 10.1371/journal.pone.0150692 (PMC4786213; doi:10.1371/journal.pone.0150692)
Supplement: S7 Table — (DOCX) [file pone.0150692.s013.docx]

**S7 Table.** **14 SNPs from additional scaffolds of the reference genome, *P. trichocarpa*.**

| Female map | |  | Male map | |  | Ref. Chromosome |
| --- | --- | --- | --- | --- | --- | --- |
| SNP | DLG |  | SNP | SLG |  |  |
| S47_114991 | 1 |  | S47_115077 | 1 |  | 1 |
| S36_177932 | 2 |  |  |  |  | 2 |
| S36_237373 | 2 |  |  |  |  | 2 |
| S34_33150 | 13 |  |  |  |  | 13 |
| S41_211942 | 17 |  | S41_211951 | 17 |  | 17 |
| S61_52548 | 17 |  |  |  |  | 17 |
| S20_619635 | 19 |  | S20_616859 | 19 |  | 19 |
| S25_413098 | 19 |  | S25_47132 | 19 |  | 19 |
|  |  |  | S25_98451 | 19 |  | 19 |
|  |  |  | S25_183156 | 19 |  | 19 |
