# Supplementary material for: Construction of High-Density Linkage Maps of Populus deltoides × P. simonii Using Restriction-Site Associated DNA Sequencing
Source: PLoS One. 2016 Mar 10;11(3):e0150692. doi: 10.1371/journal.pone.0150692 (PMC4786213; doi:10.1371/journal.pone.0150692)
Supplement: S8 Table — (DOCX) [file pone.0150692.s014.docx]

**S8 Table.** **Linkage group lengths (cM) with different mapping methods for the parental genetic maps of *P. deltoides* and *P. simonii*.**

| *P. deltoids* ‘I-69’ | | | | |  | *P. simonii* ‘L-3’ | | | | |
| --- | --- | --- | --- | --- | --- | --- | --- | --- | --- | --- |
| Group | SNP number | SAGD*^a^* | ML*^b^* | REG*^c^* |  | Group | SNP number | SAGD | ML | REG |
| DLG1 | 206 | 505.15 | 523.76 | 223.39 |  | SLG1 | 100 | 437.58 | 483.44 | 286.45 |
| DLG2 | 125 | 329.32 | 351.12 | 139.18 |  | SLG2 | 70 | 273.33 | 280.37 | 169.22 |
| DLG3 | 103 | 259.12 | 277.75 | 122.60 |  | SLG3 | 55 | 229.09 | 230.29 | 138.33 |
| DLG4 | 87 | 228.02 | 235.88 | 119.57 |  | SLG4 | 63 | 172.84 | 179.24 | 91.51 |
| DLG5 | 99 | 254.53 | 271.51 | 71.54 |  | SLG5 | 70 | 260.96 | 261.29 | 141.31 |
| DLG6 | 120 | 354.98 | 369.61 | 142.98 |  | SLG6 | 79 | 292.60 | 297.01 | 173.50 |
| DLG7 | 58 | 160.01 | 168.91 | 94.04 |  | SLG7 | 36 | 161.42 | 165.74 | 103.07 |
| DLG8 | 109 | 220.67 | 237.01 | 104.76 |  | SLG8 | 57 | 225.95 | 228.55 | 123.38 |
| DLG9 | 90 | 210.65 | 221.46 | 83.61 |  | SLG9 | 44 | 171.96 | 176.72 | 93.12 |
| DLG10 | 126 | 221.09 | 241.05 | 115.01 |  | SLG10 | 66 | 248.79 | 249.88 | 149.60 |
| DLG11 | 53 | 184.97 | 190.68 | 97.35 |  | SLG11 | 25 | 137.77 | 142.40 | 107.32 |
| DLG12 | 56 | 159.27 | 167.62 | 88.44 |  | SLG12 | 37 | 133.87 | 138.01 | 96.85 |
| DLG13 | 51 | 199.87 | 203.74 | 84.79 |  | SLG13 | 47 | 155.71 | 161.50 | 100.94 |
| DLG14 | 92 | 236.46 | 249.72 | 86.38 |  | SLG14 | 34 | 161.91 | 162.79 | 106.36 |
| DLG15 | 64 | 151.74 | 164.37 | 78.70 |  | SLG15 | 40 | 156.39 | 158.34 | 85.93 |
| DLG16 | 37 | 115.32 | 122.51 | 75.38 |  | SLG16 | 11 | 91.50 | 93.01 | 82.25 |
| DLG17 | 50 | 128.72 | 136.71 | 59.91 |  | SLG17 | 37 | 135.49 | 136.76 | 93.13 |
| DLG18 | 37 | 122.30 | 126.10 | 93.33 |  | SLG18 | 34 | 164.57 | 165.93 | 107.74 |
| DLG19 | 28 | 122.00 | 123.21 | 78.44 |  | SLG19 | 26 | 130.42 | 134.39 | 103.28 |
| DLG20 | 10 | 84.93 | 84.41 | 32.61 |  | SLG20 | 9 | 38.09 | 39.79 | 9.78 |
| Total | 1601 | 4249.12 | 4467.12 | 1992.01 |  |  | 940 | 3816.24 | 3885.43 | 2363.07 |

*^a^*SAGD: sum of adjacent genetic distances of the best orders, the same as the linkage group length in Table 3.

*^b^*ML: linkage group distances estimated from the maximum likelihood mapping algorithm of JoinMap 4.1.

*^c^*REG: linkage group distances estimated from the regression mapping algorithm of JoinMap 4.1.
